# Development and Validation of a Raman Spectroscopic Classification Model for Cervical Intraepithelial Neoplasia (CIN)

**DOI:** 10.3390/cancers14071836

**Published:** 2022-04-06

**Authors:** Damien Traynor, Shiyamala Duraipandian, Ramya Bhatia, Kate Cuschieri, Prerna Tewari, Padraig Kearney, Tom D’Arcy, John J. O’Leary, Cara M. Martin, Fiona M. Lyng

**Affiliations:** 1 Centre for Radiation and Environmental Science, FOCAS Research Institute, Technological University Dublin, D02 HW71 Dublin, Ireland; damien.traynor@tudublin.ie (D.T.); shiyamala.duraipandian@tudublin.ie (S.D.); 2 School of Physics & Clinical & Optometric Sciences, Technological University Dublin, Grangegorman, D07 XT95 Dublin, Ireland; 3 Scottish HPV Reference Laboratory, Department of Laboratory Medicine, NHS Lothian, 51 Little France Crescent, Edinburgh EH16 5SA, UK; ramya.bhatia@ed.ac.uk (R.B.); kate.cuschieri@nhslothian.scot.nhs.uk (K.C.); 4 HPV Research Group, Centre for Reproductive Health, Queens Medical Research Institute, University of Edinburgh, 47 Little France Crescent, Edinburgh EH16 4TJ, UK; 5 Discipline of Histopathology, University of Dublin Trinity College, D08 NHY1 Dublin, Ireland; prerna.tewari@tcd.ie (P.T.); padraig.kearney@tcd.ie (P.K.); olearyjj@tcd.ie (J.J.O.); cara.martin@tcd.ie (C.M.M.); 6 CERVIVA Molecular Pathology Research Laboratory, The Coombe Women and Infants University Hospital, D08 XW7X Dublin, Ireland; 7 The Trinity St. James’s Cancer Institute, D08 NHY1 Dublin, Ireland; 8 Department of Obstetrics and Gynaecology, The Coombe Women and Infants University Hospital, D08 XW7X Dublin, Ireland; tomdarcy@coombe.ie

**Keywords:** Raman spectroscopy, cytology, ThinPrep, exfoliated cells, cervical precancer, cervical intraepithelial neoplasia (CIN), HPV

## Abstract

**Simple Summary:**

There is an unmet clinical need for new methods to aid clinicians in the early detection of cervical cancer and precancer. Spectroscopic methods such as Raman spectroscopy can provide a rapid, label-free and nondestructive measurement of the biochemical fingerprint of cells, tissues and biofluids. This study aims to demonstrate the clinical utility of Raman spectroscopy for the identification of cervical precancer. Raman spectra were recorded from cervical smear samples (*n* = 662) and a classifier was developed based on histology. A classification accuracy of 91.3% was achieved in an independent blinded test set (*n* = 69), demonstrating the potential clinical utility of Raman spectroscopy.

**Abstract:**

The mortality associated with cervical cancer can be reduced if detected at the precancer stage, but current methods are limited in terms of subjectivity, cost and time. Optical spectroscopic methods such as Raman spectroscopy can provide a rapid, label-free and nondestructive measurement of the biochemical fingerprint of a cell, tissue or biofluid. Previous studies have shown the potential of Raman spectroscopy for cervical cancer diagnosis, but most were pilot studies with small sample sizes. The aim of this study is to show the clinical utility of Raman spectroscopy for identifying cervical precancer in a large sample set with validation in an independent test set. Liquid-based cervical cytology samples (*n* = 662) (326 negative, 200 cervical intraepithelial neoplasia (CIN)1 and 136 CIN2+) were obtained as a training set. Raman spectra were recorded from single-cell nuclei and subjected to a partial least squares discriminant analysis (PLSDA). In addition, the PLSDA classification model was validated using a blinded independent test set (*n* = 69). A classification accuracy of 91.3% was achieved with only six of the blinded samples misclassified. This study showed the potential clinical utility of Raman spectroscopy with a good classification of negative, CIN1 and CIN2+ achieved in an independent test set.

## 1. Introduction

Cervical cancer is the fourth most commonly diagnosed cancer, with 570,000 cases, and the fourth leading cause of cancer death, with 311,000 deaths, among women worldwide [1]. The World Health Organisation launched a global initiative in 2018 to eliminate cervical cancer by requiring an HPV vaccination, scaling up of cervical cancer screening and effective treatment [2]. Currently, there are three HPV vaccines available, a bivalent vaccine targeting high-risk HPV16 and HPV18, which account for about 70% of cervical cancer cases, a quadrivalent vaccine targeting HPV16, HPV18 and low-risk HPV6 and HPV11 and a nonavalent vaccine targeting HPV16, HPV18, HPV6, HPV11 and five other high-risk types, HPV31, HPV33, HPV45, HPV52 and HPV58, which account for another 20% of cervical cancer cases [3]. These vaccines have been shown to have excellent efficacy against cervical precancer lesions [3,4]. However, in low and middle-income countries, which have 80% of the global cancer cases, vaccination levels are low [5]. In addition, as HPV vaccination does not protect against all high-risk HPV types, high-quality screening programmes are still crucial to prevent cervical cancer [6]. Furthermore, as HPV vaccination increases and the prevalence of HPV infection decreases, the performance of current screening and triage tests will decrease. Thus, there could be a need to develop new objective tools for screening future vaccinated populations.

Recently, human papillomavirus (HPV) testing has replaced cytology for primary screening in many countries due to a higher sensitivity than cytology for the detection of high-grade cervical precancer (cervical intraepithelial neoplasia (CIN)2+) [7]. However, HPV DNA testing has a lower specificity than cytology, so additional triage tests are required to clinically manage HPV-positive women to limit over-referral to colposcopy and overtreatment [8].

Recently, optical spectroscopic techniques, such as Raman spectroscopy, have shown great promise for cancer diagnosis using tissues, cells and biofluids [9,10]. Raman spectroscopy is based on inelastic light scattering and provides a rapid, label free and nondestructive measurement of the chemical fingerprint of a sample with contributions from nucleic acids, proteins, lipids and carbohydrates. Several studies have shown the potential of this technique for cervical precancer and cancer diagnosis based on changes to the biochemistry of cervical tissues and cells [11,12,13,14,15,16,17]. In addition, both Raman spectroscopy [18,19,20] and surface-enhanced Raman spectroscopy (SERS) using gold or silver nanoparticles [21,22,23,24] have shown good potential for cervical cancer detection using blood serum or plasma. Apart from studies on pellets of exfoliated cells [25,26] and our own studies on single exfoliated cells [13,15,17,27,28,29], there have been relatively few cytology studies using Raman spectroscopy. Recently, a SERS analysis was applied to cervical exfoliated cells to discriminate normal, high-grade precancer (HSIL) and cervical squamous cell carcinoma [30].

Our group developed methods to record Raman spectra from clinical ThinPrep cytology samples on glass slides and to address blood contamination that can obscure spectral features and lead to variability [13,15,27]. We also showed that, rather than having to locate the rare abnormal cells on the unstained slide, spectra can be recorded from any cells [28]. This is because biochemical changes can be detected in morphologically normal appearing cells due to a field change in the whole cervical epithelium at a biochemical level. We recently published a protocol for Raman spectral cytopathology, which covers sample preparation, spectral acquisition, preprocessing and data analysis [31].

To date, however, the studies used small sample sizes and the results were validated using leave-one-out cross validation rather than using an independent test set. The aim of the present study is to show the clinical utility of Raman spectroscopy for identifying cervical precancer by developing a classification model based on spectral data from negative, CIN1 and CIN2+ ThinPrep cytology samples (*n* = 662) and by validating this model by means of an independent blinded dataset (*n* = 69).

## 2. Materials and Methods

### 2.1. Sample Collection

Cervical smear samples collected in PreservCyt solution were obtained with consent from patients attending the Colposcopy clinic at the Coombe Women and Infants University Hospital (CWIUH), Dublin, Ireland (*n* = 390). Ethical approval for use of pseudo-anonymised samples for this study was granted by the CWIUH Research Ethics Committee (study no. 28–2014).

A further set of cervical smear samples (*n* = 392) was provided through application to the Scottish HPV Archive, a research tissue biobank set up to facilitate HPV-associated research. The archive comes under the auspice of the National Research for Scotland tissue bioresource, 20/ES/0061. Our previous study demonstrated that samples stored at −25 °C could be analysed successfully by Raman spectroscopy and that fresh samples, biobanked (stored) samples and pooled fresh and biobanked samples achieved a similar sensitivity and specificity for detection of CIN 2+ [17]. Biobanked LBC samples used for this study were sedimented with the cellular pellet transferred into a 4.5 mL vial for long-term storage at −25 °C in PreservCyt. After transit, samples were reconstituted to a volume of 20 mL fresh PreservCyt solution to resemble the original LBC specimen from which the sample was derived.

Samples were collected from each patient according to similar standard operating procedures issued by CervicalCheck, Ireland’s National Cervical Cancer Screening Programme, and the NHS Scottish Cervical Screening Programme.

**Figure 1:**
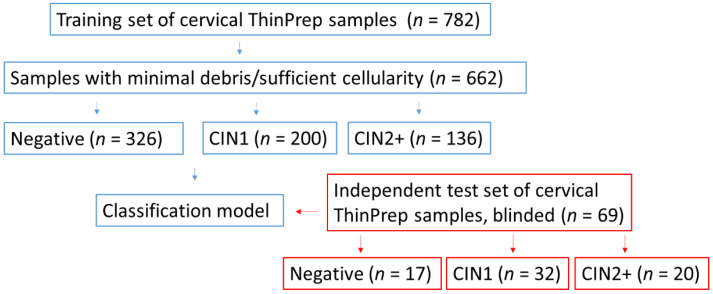
Schematic showing an overview of the training set and the independent test set of cervical ThinPrep samples.

Of the 782 samples obtained for the study, 662 samples were suitable for Raman analysis and comprised the training set. Excluded samples (*n* = 120, 15%) had insufficient cells remaining in the ThinPrep vial after cytology and HPV tests were performed or had excessive debris covering any remaining cells. These samples could not be used for Raman spectroscopic analysis and had to be excluded. The training set consisted of samples which were either confirmed as normal by histology with no abnormality detected (*n* = 115) or were negative on cytology and/or colposcopy but had no biopsy or histology (*n* = 211), samples which were confirmed as CIN1 by histology (*n* = 200) and samples which were confirmed as CIN2 or CIN3 (CIN2/3) by histology (*n* = 136) (Figure 1). Samples which were negative on cytology and/or colposcopy but which had no biopsy or histology result were combined with the samples confirmed as normal by histology to create a ‘negative’ set of *n* = 326 samples.

An additional set of blinded samples (*n* = 69) was provided by the Scottish HPV Archive as above. These samples were used as an independent test set to validate the classification model. This set of samples was blinded to the researchers until after the spectral acquisition, data preprocessing and analysis steps were carried out. The samples were confirmed by histology as normal (*n* = 17), CIN1 (*n* = 32) and CIN2+ (*n* = 20) (Figure 1).

### 2.2. HPV Testing

Cervical smear samples were tested for HPV using a variety of technologies. Cervical smear samples collected by the CERVIVA team at the Coombe Women and Infants University Hospital were tested for HPV DNA using the Cobas HPV DNA test (Roche) and for HPV mRNA using the Aptima HPV Assay (Hologic), as part of a series of CERVIVA research programmes underway at that time.

Samples collected as part of the Scottish HPV archive were tested for HPV using a variety of tests, including: Hybrid Capture 2 (HC2) (Qiagen), Cobas HPV Assay (Roche), Cepheid Expert HPV assay, the Abbott Real-Time HPV Assay, the Aptima HPV Assay (Hologic) and Optiplex HPV Genotyping test (Diamex, GMBH).

The Cobas HPV test (Roche) is a fully automated PCR-based HPV DNA test that detects in three separate channels: HPV16 individually, HPV18 individually and a pool of 12 other HPV genotypes (11 definite high-risk genotypes plus 1 possibly high-risk genotype) as follows: HPV 31, 33, 35, 39, 45, 51, 52, 56, 58, 59, 66 and 68.

The Aptima HPV Assay (Hologic) detects mRNA coding for the E6 and E7 viral proteins of HPV types 16, 18, 31, 33, 35, 38, 39, 45, 51, 52, 56, 58, 59, 66 and 68. The Aptima HPV Assay involves three main steps, which take place in a single tube: target capture, target amplification by transcription-mediated amplification (TMA) and detection of the amplification products (amplicon) by the hybridization protection assay (HPA). A 1ml aliquot of PreservCyt specimen was used.

The HC2 High-Risk HPV DNA test (Qiagen) uses Hybrid Capture^®^2 technology which is a nucleic acid hybridization assay with signal amplification that utilizes microplate chemiluminescent detection. Specimens containing the target DNA hybridize with a specific HPV RNA probe cocktail. The HC2 High-Risk HPV DNA Test detects HPV DNA from 13 high-risk HPV types, 16, 18, 31, 33, 35, 39, 45, 51, 52, 56, 58, 59 and 68.

The XPert HPV Assay (Cepheid) is a real-time polymerase chain reaction (PCR) assay for the detection of 14 high-risk types of HPV DNA through five separate channels (HPV16, HPV18 and 45, HPV31, 33, 39, 52 and 58, HPV51 and 59 and HPV39, 56, 66 and 68). The assay is formulated in a single-use cartridge, provides a result within one hour, can be performed by non-laboratory-trained health-care workers and requires minimal hands-on time.

The Abbott Real-Time HPV Assay (Abbott) detects 14 high-risk HPV genotypes with simultaneous identification of HPV 16 and HPV 18.

The Optiplex HPV Genotyping test (Diamex, GMBH) is a multiplex in vitro test kit based on Luminex Technology for the qualitative determination of human papillomavirus (HPV) genotypes 6, 11, 16, 18, 26, 31, 33, 35, 39, 42, 43, 44, 45, 51, 52, 53, 56, 58, 59, 66, 68, 70, 73 and 82 in polymerase chain reaction (PCR)-amplified samples of genomic DNA isolated from cervical smears.

The Roche Linear Array HPV Genotyping Test is a qualitative test that detects 37 high- and low-risk human papillomavirus genotypes.

### 2.3. ThinPrep Slide Preparation

Samples were prepared for Raman spectroscopy using the ThinPrep 2000 processor (Hologic Inc., Marlborough, MA, USA). The ThinPrep processor homogenized the sample by spinning the filter, creating shear forces that break up any clumped material (blood, mucin and nondiagnostic material). The cells were then transferred onto the TransCyt filter and transferred onto a glass slide to produce a monolayer of cells approx. 20 mm in diameter. The slide was then ejected into a fixative bath of 95% ethanol.

The slides then underwent a pretreatment step to remove any molecular contamination by haemoglobin, which obscured several features of the cellular spectrum as described in our published protocol [31]. Briefly, slides were treated with a 30% solution of H_2_O_2_ at room temperature for 3 min, followed by a 70% solution of industrial methylated spirits (IMS) for 3 min followed by multiple dips into 100% IMS to remove any remaining cellular debris and H_2_O_2_ and were air dried.

### 2.4. Raman Spectroscopy

Raman spectra were acquired as described in our published protocol [31] using a HORIBA Jobin Yvon XploRA system (Villeneuve d’Ascq, France), which incorporates an Olympus microscope BX41 equipped with a X100 objective (MPlan, Olympus, NA = 0.9). A 532 nm diode laser source was used. Laser power was set to 100%, resulting in 16 mW at the objective. The confocal hole coupled to a slit aperture of 100 μm was set at 100 μm for all the measurements. The resultant Raman signals were detected using a spectrograph with a 1200 g∕mm grating coupled to a charge-coupled device (Andor, 1024 × 256 pixels). The spectrometer was controlled by Labspec V6.0 software. For each cell, a Raman spectrum was acquired from the nucleus in the fingerprint region, 400 to 1800 cm^−1^, with an integration time of 30 s averaged over two accumulations. Spectra were recorded only from the cell nucleus as these were found to be more reproducible and consistent than spectra from the cell cytoplasm [27]. Where possible, spectra were recorded from at least 20–30 randomly selected morphologically normal superficial and intermediate cells from each unstained Pap smear.

### 2.5. Data Preprocessing and Analysis

Data were normalised and analysed using MATLAB software (MathWorks) and specific scripts developed and adapted for uploading of the spectra and their preprocessing, including smoothing (Savitzky–Golay K = 5, K = 13), baseline correction (rubberband) and vector normalization. The spectra were corrected for the glass background using a linear least-squares method with non-negative constraints (NNLS) as described in our published protocol [31]. The data were mean centred and subjected to partial least squares discriminant analysis (PLS-DA) using the PLS toolbox (Eigenvector Research, Washington, DC, USA) in the MATLAB (MathWorks Inc., Natick, MA, USA) environment. PLS-DA has the ability to distinguish between known classifications of samples and its aim is to find latent variables (LVs) and directions to maximise separation in a multivariate space. In this study, PLS-DA was used to build a classification model from a training set of data (*n* = 662) and a blinded test set (*n* = 69) was employed to validate the model.

## 3. Results

### 3.1. Training Set

Table 1 shows a summary of the training set samples according to cytology, HPV DNA and HPV mRNA results. Supplementary Tables S1 and S2 show the details of the samples obtained from CWIUH and the Scottish HPV archive, respectively.

### 3.2. Training Set—Cytology

Cytology results were available for over 95% of the training set samples and are shown in Table 1. The normal samples were either negative (34.97%, *n* = 114), borderline (BNA) (26.38%, *n* = 86) or low-grade (LSIL) (29.14%, *n* = 95) on cytology, with low numbers of samples with atypical (ASCUS/ASC-H/ASC-BNA) (1.53%, *n* = 5), high-grade (HSIL) (3.07%, *n* = 10) or no cytology results (4.91%, *n* = 16). Almost half of the CIN1 samples were LSIL (49%, *n* = 98) and almost a third were BNA (27.5%, *n* = 55) on cytology. The remainder of the CIN1 samples were HSIL (9.5%, *n* = 19), negative (5.5%, *n* = 11), ASCUS/ASC-H/ASC-BNA (5.5%, *n* = 9) or had no cytology results (3%, *n* = 6). The CIN2+ samples were either LSIL (30.88%, *n* = 42), HSIL (28.68%, *n* = 39) or BNA (19.85%, *n* = 27) on cytology, with low numbers of samples with negative (5.15%, *n* = 7), ASCUS/ASC-H/ASC-BNA (6.62%, *n* = 9), high-grade/invasive (2.94%, *n* = 4) or no cytology results (5.88%, *n* = 8).

### 3.3. Training Set—HPV Testing

HPV DNA test results (hc2, Linear Array, Cobas, Optiplex, Luminex, Abbott or Cepheid test) were only available for less than half of the training set samples (45.46%, *n* = 301) and are shown in Table 1. For the normal samples, 38.34% had an HPV DNA test result (*n* = 125), and of these, 45.6% had an HPV DNA-negative result (*n* = 57), 24% had an HPV DNA-positive result (*n* = 30) according to an hc2 or Linear Array test and 30.4% had an HPV genotype result from a Cobas, Optiplex, Luminex, Abbott or Cepheid test (*n* = 38). The majority were either HPV16 and other high-risk HPV types (*n* = 10) or other high-risk HPV types (*n* = 21) (Table 1). Less than half of the CIN1 samples had an HPV DNA test result (46.5%, *n* = 93), and of these, 8.6% had a negative result (*n* = 8), 32.25% had a positive result (*n* = 30) according to an hc2 or Linear Array test and 59.14% had an HPV genotype test result (Cobas, Optiplex, Luminex, Abbott or Cepheid), (*n* = 55). The majority were HPV16 and other high-risk HPV types (*n* = 11) or other high-risk HPV types (*n* = 28) (Table 1). More than 60% of the CIN2+ samples had an HPV DNA test result (61.02%, *n* = 83), and of these, no samples had a negative result, 21.68% had a positive result (*n* = 18) according to an hc2 or Linear Array test and 78.31% had an HPV genotype result from a Cobas, Optiplex, Luminex, Abbott or Cepheid test (*n* = 65). Again, the majority were HPV16 and other high-risk HPV types (*n* = 23) or other high-risk HPV types (*n* = 22) (Table 1).

HPV mRNA test results (Aptima) were available for 35.65% of the training set samples (*n* = 236) and are shown in Table 1. For the normal samples, 32.82% had an HPV mRNA test result (*n* = 107), and of these, 55.14% had a negative result (*n* = 59) and 44.86% had a positive result (*n* = 48). Less than half of the CIN1 samples had an HPV mRNA test result (46.5%, *n* = 93), and of these, 27.96% had a negative result (*n* = 26) and 73.12% had a positive result (*n* = 68). Only 25.74% of the CIN2+ samples had an HPV mRNA test result (*n* = 35), and of these, 20% had a negative result (*n* = 7) and 80% had a positive result (*n* = 28).

### 3.4. Training Set—Raman Spectroscopy

**Figure 2:**
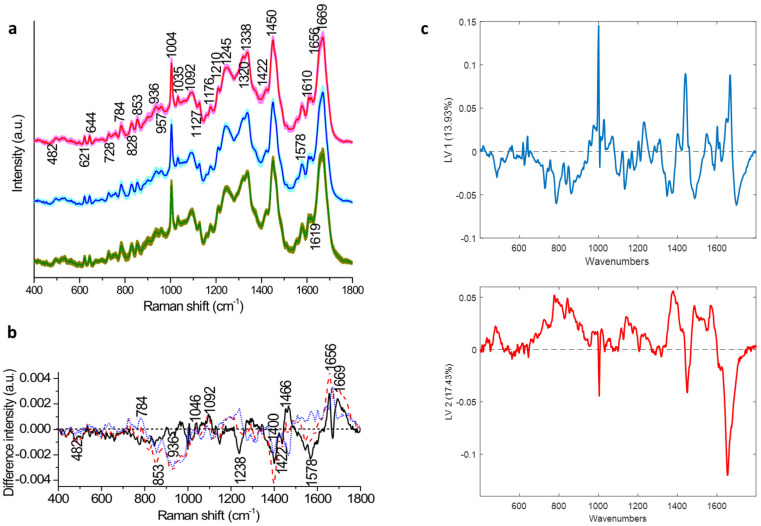
(**a**) Mean Raman spectra of negative (green), CIN1 (blue) and CIN2+ (red) samples; shading indicates the standard deviation; (**b**) Different Raman spectra of negative (CIN1) (black), negative (CIN2+) (red), and CIN1-CIN2+ (blue); (**c**) partial least squares (PLS) and latent variables (LV1 and LV2) of the PLS-DA model developed from the training set.

The mean Raman spectra recorded from the negative, CIN1 and CIN2+ samples are shown in Figure 2a. Peaks were evident at 482 cm^−1^ (glycogen), 621 and 644 cm^−1^ (proteins), 728 and 784 cm^−1^ (DNA), 828 cm^−1^ (DNA/RNA), 853 and 936 cm^−1^ (glycogen and proteins), 957 cm^−1^ (DNA), 1004 cm^−1^ (phenylalanine), 1035 cm^−1^ (proteins), 1092 cm^−1^ (DNA phosphate backbone), 1127 cm^−1^ (proteins), 1176 cm^−1^ (cytosine/guanine), 1210 cm^−1^ (tryptophan and phenylalanine), 1245 cm^−1^ (amide III), 1320 cm^−1^ (DNA/RNA, proteins and amide III), 1338 cm^−1^ (glycogen, proteins and nucleic acids), 1422 cm^−1^ (DNA/RNA), 1450 cm^−1^ (proteins and lipids), 1578 cm^−1^ (nucleic acids), 1610 cm^−1^ (phenylalanine and tyrosine), 1656 and 1669 cm^−1^ (amide I) (Table 2). Difference spectra between negative and CIN1 samples, negative and CIN2+ samples and CIN1 and CIN2+ samples are shown in Figure 2b. The different spectra exhibited positive peaks at 784, 1046, 1092 (nucleic acids), 1466, 1656 and 1669 cm^−1^ (proteins) and negative peaks at 482, 853, 936 (glycogen), 1238 (proteins), 1400, 1422 and 1578 cm^−1^ (nucleic acids).

A PLSDA classification model was developed from the spectral data of the training set. The PLSDA latent variables (LVs) showed similar features to those observed in the difference spectra at 482, 728, 784, 828, 852, 1092, 1238, 1334, 1380, 1450, 1485, 1578, 1656 and 1669 cm^−1^.

### 3.5. Independent Test Set

The next step was to validate the results with an independent test set. A further set of 69 samples was obtained from the Scottish HPV Archive and these comprised the validation set for the study. This set of samples was blinded to the researchers until after the spectral acquisition, data preprocessing and analysis steps were carried out. The samples were confirmed by histology as normal (*n* = 17), CIN1 (*n* = 32) and CIN2+ (*n* = 20) (Figure 1, Table 3).

### 3.6. Independent Test Set—Cytology

Cytology results were available for over 98% of the validation set samples and are shown in Table 3. The normal samples were either low-grade (70.59%, *n* = 12) or borderline (29.41%, *n* = 5) on cytology. Similarly, the CIN1 samples were mainly low-grade (78.13%, *n* = 25) or borderline (15.63%, *n* = 5), with low numbers of high-grade (3.13%, *n* = 1) or no cytology result (3.13%, *n* = 1). The CIN2+ samples were a mixture of negative (30%, *n* = 6), borderline (30%, *n* = 6), low-grade (10%, *n* = 2) and high-grade (30%, *n* = 6) cytology.

### 3.7. Independent Test Set—HPV Testing

HPV DNA test results (Luminex test for *n* = 68 samples and additional Abbott and BD tests for *n* = 8 samples) were available for over 98% of the validation set samples and are shown in Table 3. The normal samples were 70.59% other high-risk HPV types (*n* = 12), 17.65% HPV16 and other high-risk HPV types (*n* = 3), 5.88% low-risk HPV types (*n* = 1) and 5.88% no HPV types (*n* = 1). Similarly, the CIN1 samples were 68.75% other high-risk HPV types (*n* = 22), 18.75% HPV16 and other high-risk HPV types (*n* = 6), 3.13% low-risk HPV types (*n* = 1) and 3.13% no HPV types (*n* = 1) (Table 3). The HPV-positive CIN2+ samples were a mixture of other high-risk HPV types (47.37%, *n* = 9), HPV16 and other high-risk HPV types (21.05%, *n* = 4), HPV16 (10.53%, *n* = 2), HPV16, 18 and other high-risk HPV types (5.26%, *n* = 1) and no HPV types (15.79%, *n* = 3).

HPV mRNA test results (Aptima) were available for 56.52% of the validation set samples (*n* = 39) and are shown in Table 3. Of the normal samples tested, 100% had a positive result (*n* = 5). Of the CIN1 samples tested, 33.33% had a negative result (*n* = 5) and 66.66% had a positive result (*n* = 10). Of the CIN2+ samples tested, 31.57% had a negative result (*n* = 6) and 68.42% had a positive result (*n* = 13).

### 3.8. Independent Test Set—Raman Spectroscopy

The validation results generated using the blinded test set (*n* = 69) are shown in the confusion matrix (Table 4). All of the negative samples (17/17), 30/32 of the CIN1 samples and 16/20 of the CIN2+ samples were classified correctly. The Raman classification results, together with the histology, cytology, HPV DNA and HPV mRNA results, for the six misclassified samples are shown in Table 5. The misclassified samples were all classified incorrectly as negative by our classification model. Two of the samples were histology confirmed CIN1 and were low-grade on cytology, positive for other high-risk HPV types (HPV 51 and HPV 52) and positive for HPV mRNA. The other four samples were histology confirmed CIN2+. Three of these CIN2+ samples were positive for other high-risk HPV types (HPV 33, 52, 56), positive for HPV mRNA and one sample was low-grade on cytology, while the other two were high-grade on cytology. The final CIN2+ sample was negative on cytology, had no HPV types and was negative for HPV mRNA.

## 4. Discussion

The use of Raman spectroscopy for the classification of cervical exfoliated cells has been demonstrated previously [13,15,16,17], but an independently validated Raman classification model for negative, CIN1 and CIN2+ ThinPrep samples was not published before.

Initially, Raman spectra were recorded from a training set consisting of cervical ThinPrep samples (*n* = 662). The different spectra and latent variables (LV1 and LV2) showed that the discrimination was mostly based around increased nucleic acids (728, 784, 828, 1092, 1485 and 1578 cm^−1^), decreased glycogen (482, 852, 936, 1334 and 1380 cm^−1^) and changes in protein features (1238, 1450, 1656 and 1669 cm^−1^), indicating an increased proliferation and altered protein expression as a result of HPV infection. These discriminating features were consistent with our previous studies [13,17,27,28,29].

A PLSDA classification model was developed from the training set for discrimination between negative, CIN1 and CIN2+ ThinPrep samples, and validated using an independent blinded dataset (*n* = 69). Raman spectra were recorded from this test set and used to test the classification model. In total, 63/69 samples (91.30%) were classified correctly. All 17 negative samples were classified correctly, but 2 CIN1 samples and 4 CIN2+ samples were incorrectly classified as negative. Five of the misclassified samples were low-grade or high-grade on cytology and were high-risk HPV DNA-positive and HPV mRNA-positive. Interestingly, one of the misclassified samples, which was CIN3 on histology, was negative on cytology, had no HPV types and was HPV mRNA-negative. Although misclassified according to histology results, interestingly, the negative classification was consistent with the cytology and HPV test results. Our study showed similar performance to a recently reported SERS approach using gold nanoparticle substrates to enhance the Raman signal of cervical exfoliated cells [30]. In the SERS study, accuracies of 94.46%, 71.6% and 97.72% were achieved for single exfoliated cells, cell pellets and extracted DNA, respectively. High accuracies were achieved for extracted DNA for normal, high-grade precancer and cancer samples, whereas for the single cells and cell pellets, high accuracies were achieved for normal and cancer samples, but not for the high-grade samples. Our study used label-free Raman spectroscopy to measure the biochemical signature of exfoliated cells and achieved a good classification of normal, low-grade and high-grade precancer samples.

In our study, for the samples with CIN1 histology, the test set corresponded reasonably well to the training set, as both sets of samples were mainly comprised of samples with borderline or low-grade cytology. For the samples with CIN2+ histology, the training set mostly consisted of samples with borderline, low-grade and high-grade cytology, while the test set also contained samples with negative cytology (*n* = 6, 30%). For the samples with normal histology, these were comprised of samples with negative, borderline and low-grade cytology for the training set, but only borderline and low-grade cytology for the test set. As not all samples were tested for HPV DNA and mRNA, it was difficult to compare the training and test sets in terms of HPV test results. Another limitation of the study was that not all of the negative samples in the training set were confirmed by histology as normal, but this was difficult to achieve, as a biopsy was not performed for all patients undergoing a colposcopy. In addition, the sample set represented a disease-enriched population to increase the number of disease cases. This is standard practice when evaluating a new technology, particularly in the case of cervical cancer, where CIN2 prevalence is only around 1% in routinely screened high-income countries. Future work should involve a prospective study in a screening population. Although a technical validation exercise was performed demonstrating that stored samples are credible biospecimens for Raman analysis [17], the use of “fresher” samples may be associated with enhanced performance and this will be explored in a future prospective study.

Recently, HPV testing has been introduced for primary cervical screening, but the HPV DNA test cannot distinguish between a transient and a transforming HPV infection, and cytology-based triage is also needed. Our study aimed to evaluate Raman spectroscopy as a tool to support current cervical screening approaches, HPV testing and cytology. As HPV primary screening has been implemented widely, there is a need to improve the specificity through the use of a good triage test. Raman spectroscopy may be a good alternative to cytology triage, as it is less subjective with a better sensitivity. Our recent study has shown that Raman spectroscopy has potential as a triage test for HPV-positive women to identified transforming HPV infections [36], although further work is necessary on a larger sample size and in a screening population where HPV is the primary screening test.

In addition, in the future, with more widespread HPV vaccination, the performance of techniques relying on a subjective assessment, such as cytology, should decrease as a consequence of the reduction in the prevalence of the disease. Thus, new objective tools, such as Raman spectroscopy, would be needed for screening future vaccinated populations. Raman spectroscopy is a low-cost solution that has the potential to be integrated into existing services, as well being a potential solution for developing countries, where “see-and-treat” options are preferred to increase the effectiveness of screening hard-to-reach populations.

## 5. Conclusions

In this study, a classification model for negative, CIN1 and CIN2+ ThinPrep samples was developed and validated. The model was developed from a training set of 662 samples and an accuracy of 91.3% was achieved in an independent test set of 69 samples.

Raman spectroscopy is a label-free objective tool that may be a good alternative to cytology for the triage of HPV-positive cases. It may also be important in the future as HPV vaccination increases, and the prevalence of the disease decreases, because the reduced performance of current screening and triage tests should result in a clinical need for new objective tools for screening vaccinated populations.

## Figures and Tables

**Table 1:** Summary of training set of samples, including cytology, HPV DNA and HPV mRNA results.

Test		No Histology/ Normal		CIN1		CIN2+	
		326		200		136	
		*n*	%	*n*	%	*n*	%
Cytology	Negative	114	34.97	11	5.50	7	5.15
	ASCUS	1	0.31	2	1.00	0	0.00
	ASC-H	2	0.61	3	1.50	7	5.15
	ASC-BNA	2	0.61	6	3.00	2	1.47
	BNA	86	26.38	55	27.50	27	19.85
	LSIL	95	29.14	98	49.00	42	30.88
	HSIL	10	3.07	19	9.50	39	28.68
	High-grade/invasive	0	0.00	0	0.00	4	2.94
	No cytology	16	4.91	6	3.00	8	5.88
	Total	326	100	200	100	136	100
HPV DNA	HPV DNA-positive	30	9.20	30	15.00	18	13.24
	HPV DNA-negative	57	17.48	8	4.00	0	0.00
	HPV16	0	0.00	1	0.50	7	5.15
	HPV18	0	0.00	1	0.50	1	0.74
	HPV16, 18	1	0.31	0	0.00	1	0.74
	HPV16, other hrHPV types	10	3.07	11	5.50	23	16.91
	HPV18, other hrHPV types	2	0.61	4	2.00	3	2.21
	HPV16, 18, other hrHPV types	1	0.31	2	1.00	4	2.94
	Other hrHPV types	21	6.44	28	14.00	22	16.18
	Low-risk HPV types	1	0.31	4	2.00	1	0.74
	No HPV types	2	0.61	4	2.00	3	2.21
	Not tested	201	61.66	107	53.50	53	38.97
	Total	326	100	200	100	136	100
HPV mRNA	HPV mRNA positive	48	14.72	68	34.00	28	20.59
	HPV mRNA negative	59	18.10	26	13.00	7	5.15
	Not tested	219	67.18	106	53.00	101	74.26
	Total	326	100	200	100	136	100

**Table 2:** Tentative peak assignments [32,33,34,35].

Raman Peak Position (cm^−1^)	Proteins	Lipids	Carbohydrates	Nucleic Acids
482			Glycogen	
577			Glycogen	
621	C–C twist Phe			
644	C–C twist Tyr			
728	CH_2_ def	C–C head		A
784				U, C, T ring br
828	Out of Plane ring br. Tyr			PO_2_ a.str
853			Glycogen	
936			Glycogen	
1004	Sym. Ring br. Phe			
1035	C–H in plane Phe, C–C str			
1092				PO_2_
1127	C–N str	Chain C–C str	C–O str, Glycogen	
1176				C, G
1210	C–C_6_H_5_ str. Phe, Trp			
1238	C–N str, Amide III			
1338	Trp		Glycogen	G
1450	CH_2_ def	CH_2_ def		
1485	CH_2_ def			G, A
1578				A, G ring br
1610	C=C Phe, Tyr			
1656	C=O str, C = C sym. str.			
1669	C=O str. Amide I			

**Table 3:** Summary of test set of samples, including cytology, HPV DNA and HPV mRNA results.

Test		Normal		CIN1		CIN2+	
		17		32		20	
		*n*	%	*n*	%	*n*	%
Cytology	Negative	0	0.00	0	0.00	6	30.00
	Borderline squamous changes	5	29.41	5	15.63	6	30.00
	Mild dyskaryosis	12	70.59	25	78.13	2	10.00
	Moderate dyskaryosis	0	0.00	1	3.13	3	15.00
	Severe dyskaryosis	0	0.00	0	0.00	3	15.00
	High-grade/invasive	0	0.00	0	0.00	0	0.00
	No cytology	0	0.00	1	3.13	0	0.00
	Total	17	100	32	100	20	100
HPV DNA	HPV16	0	0.00	0	0.00	2	10.00
	HPV18	0	0.00	0	0.00	0	0.00
	HPV16, 18	0	0.00	0	0.00	0	0.00
	HPV16, other hrHPV types	3	17.65	6	18.75	4	6.25
	HPV18, other hrHPV types	0	0.00	1	3.13	0	0.00
	HPV16, 18, other hrHPV types	0	0.00	1	3.13	1	5.00
	Other hrHPV types	12	70.59	22	68.75	9	45.00
	Low-risk HPV types	1	5.88	1	3.13	0	0.00
	No HPV types	1	5.88	1	3.13	3	15.00
	Not tested	0	0.00	0	0.00	1	5.00
	Total	17	100	32	100	20	100
HPV mRNA	HPV mRNA positive	5	29.41	10	31.25	13	68.42
	HPV mRNA negative	0	0.00	5	15.63	6	31.58
	Not tested	12	70.59	17	53.13	1	5.00
	Total	17	100	32	100	20	105

**Table 4:** Confusion matrix showing classification of negative, CIN1 and CIN2+ samples from the test set.

Class	Negative	CIN1	CIN2+
Negative	17	0	0
CIN1	2	30	0
CIN2+	4	0	16

**Table 5:** Cytology, HPV testing and histology results and Raman classification for the misclassified samples.

Sample No.	Cytology	HPV DNA	HPV mRNA	Histology	Raman Classification
B019	Mild dyskaryosis	HPV 52	Positive	CIN 1	Negative
B020	Negative	No types	Negative	CIN 3	Negative
B033	Mild dyskaryosis	HPV 6, 52, 56	Positive	CIN 3	Negative
B035	Severe dyskaryosis	HPV 11, 33, 52	Positive	CIN 3	Negative
B036	Moderate dyskaryosis	HPV 56	Positive	CIN 3	Negative
B055	Mild dyskaryosis	HPV 51	Positive	CIN 1	Negative

## Data Availability

The data that support the findings of this study are available from the corresponding author upon reasonable request.

## Supplementary Materials

The following supporting information can be downloaded at: https://www.mdpi.com/article/10.3390/cancers14071836/s1, Table S1: Summary of sample set from CWIUH, including cytology, HPV DNA and HPV mRNA results, Table S2: summary of sample set from Scottish HPV Archive, including cytology, HPV DNA and HPV mRNA results.
